# (Dis)passionate law stories: the emotional processes of encoding narratives in court

**DOI:** 10.1111/jols.12355

**Published:** 2022-05-08

**Authors:** STINA BERGMAN BLIX, ALESSANDRA MINISSALE

**Affiliations:** ^1^ Department of Sociology Uppsala University Box 624 Uppsala 751 26 Sweden

## Abstract

In this conceptual article, we propose that legal professional decision makers’ transformation of narratives in court (encoding) influences their emotional attunement to the stories at hand. First, we argue that the process of encoding is linked to the strict demand for dispassion in legal settings. Second, we introduce three techniques that regulate the emotional processes at play during the encoding of law narratives: *demarcation*, *fragmentation*, and *proximation*. Demarcation and fragmentation produce emotional distance from narratives and their associated emotions, while proximation refers to the deliberate calibration of emotional attunement to law stories to enable legal decision making. Demarcation and fragmentation are sustained by background emotions of ease and interest when stories align with legal requirements, versus disinterest and irritation when ‘too many’ details are introduced. Proximation is regulated through the epistemic emotions of doubt and certainty. By scrutinizing the subtle emotions involved in legal encoding, we problematize the ideal of judicial dispassion.

## 1 INTRODUCTION

One important argument for the narrative turn in socio‐legal studies is to move away from a positivist understanding, a God's‐eye view,^1^ of objectivity and impartiality towards an understanding that accounts for the role of interpretation in legal evaluation and judgment.^2^ Narrative – that is, accounts or stories of events occurring over time^3^ – ‘corresponds more closely to the manner in which the human mind makes sense of experience than does the conventional, abstracted rhetoric of law’.^4^ People build mental models, using ‘narrative imagining’^5^ as a basis for analytical thinking. Thus, one attraction of the narrative turn is that it can address how embodied human beings, such as legal decision makers, make objective decisions during actual legal practice.^6^ Most research thus far has focused solely on how discursive practices influence interpretations of legal matters,^7^ leaving aside the role of emotions, another fundamental component of the human mind.

In this conceptual article, we focus on how legal professional decision makers’ transformation of narratives in court (encoding) influences their emotional attunement to the stories at hand. It is important to study legal professional decision makers because they usually decide at all court levels in civil legal systems and in bench trials^8^ and higher courts in common legal systems.^9^ First, we argue that legal encoding is influenced by the strict demand for ‘judicial dispassion’.^10^ This is not to say that emotions do not matter in legal story building,^11^ but rather that legal professionals’ approaches to stories influence the ways in which emotions matter. In contrast to lay story building, legal professional story building is restricted to a focus on facts,^12^ a feature that has been shown to attenuate empathic inclination towards the people in a story.^13^ Second, we introduce three techniques that regulate emotional attunement to the narratives told and evaluated in court for the purposes of legal encoding: *demarcation* refers to the strict temporal and content boundaries of law stories; *fragmentation* denotes the dissected way in which stories are presented; and *proximation* regulates empathic perspective taking and emotional attunement to the stories at hand. Using illustrations from judgments, observation extracts, and interview data from an ongoing research project, we argue that demarcation and fragmentation produce emotional distance from narratives and their associated emotions by directing interest towards details in the stories relevant to legal encoding, and disinterest or irritation when facets outside this realm are introduced. While demarcation and fragmentation regulate emotional attunement to stories by indirect means, proximation refers to the deliberate calibration of emotional attunement to stories at hand and their emotional content, with a view to enabling and augmenting legal decision making.

In the next section, we discuss narratives in a legal institutional frame, introducing the techniques of demarcation and fragmentation to explore the transformation of stories into legal categories. Thereafter, we present our sociological perspective on emotion, centring on the emotional dynamics of dispassionate justice. Next, we propose how the techniques of demarcation, fragmentation, and proximation foster and regulate emotions, using illustrations from real court cases to investigate professional legal decision making. In the concluding discussion, we raise issues for further research.

## 2 THE LEGAL TRANSFORMATION OF NARRATIVES AS ENCODING

Everyday stories and law stories are constructed in different ways. In court, the stories told by witnesses, victims, or defendants/litigants during examinations require translation to fit into legal categories.^14^ Prosecutors and defence lawyers need to ‘purify issues for [the court's] consideration’.^15^ This is because the encoded version of a story represents the authoritative account in court. People generally accept that encoding overrides stories, and lay and professional parties in court try to frame a story to fit those expectations. An inherent tension in this transformation is that while law stories are deductive, as they need to link a defendant to an action that caused harm to a plaintiff or victim, encoding per se is not. Legal encoding is done to ensure objective and impartial evaluation that builds on categorization, procedure, and rules^16^ rather than on causality. Legal professionals need to evaluate whether stories match the relevant criteria for fitting within a statute or precedent. This explains why law stories, which are made to facilitate encoding, are often ‘*anti*‐storylike’.^17^

We argue that two important techniques for transforming everyday stories into law stories are demarcation and fragmentation. Demarcation refers to the strict temporal and content boundaries of law narratives,^18^ while fragmentation refers to the way in which evidence is presented and argued in court in an anti‐narrative style.^19^ This may mean that evidence is presented in a non‐sequential order; that lawyers only ask about ‘isolated fragments’;^20^ or that the whole hearing is fragmented, with examinations that occur sporadically over several weeks or even months.^21^

In everyday life, stories have no natural beginning or end. The choice of where to start and end a story depends on the storyteller's relationship to the audience and the social situation in which the story is told. Law narratives, by contrast, are expected to rotate around ‘the trouble’^22^ – that is, the events leading up to the critical incident. Previous research has shown that the court often rejects attempts by defendants to widen the timeframe or add contextual factors leading up to an event.^23^ The everyday inductive style of storytelling often complicates the issue of responsibility,^24^ whereas a law story needs to establish intents and actions. The narrowing of the temporal boundaries produces actions as intentional or determinate.^25^ Demarcation and fragmentation are thus active techniques for transforming lay narratives used in court across democratic legal systems.

Counsel and judges have well‐considered reasons to limit the part lay participants play in proceedings. They restrict explanations of law and procedure, have mixed motives regarding upset (a resource for case‐making), and prefer forms of testimony that vest control in themselves rather than lay participants.^26^

As Fielding describes, legal professionals, in particular the presiding judge, control proceedings in court; they demarcate and fragment the presentation of narratives to streamline law narratives so as to enable encoding. The content of the narrative is also restricted by multiple rules depending on the legal system.^27^ The rules of evidence, which vary in strictness from country to country, hinder a flowing account, as the law requires pure, dry descriptions that ‘isolate’ the event under dispute; this also guarantees a smooth trial.^28^ That is to say, the legal frame treats stories as abstract entities from which to pick out relevant details. Before we discuss the emotional dynamics of legal encoding, we present our emotion theory framework as being linked to the persistent legal institutional demand for dispassion.

## 3 THE EMOTIONAL PROCESSES OF DISPASSIONATE JUSTICE

Western jurisdictions are governed by an ideal of judicial dispassion, which is a strong norm that expects legal professionals in general, and judges in particular, to be able to set aside potential desires to reach a particular outcome.^29^ They need to distance themselves from inflammatory and unfairly prejudicial evidence, not allowing it to warp their judgment.^30^ The association of partiality and prejudice with emotion manifests the ‘legal ideology’^31^ entailing that the autonomy of the law requires emotionless reason. In recent decades, scholars have challenged this tension between emotion and legal rationality,^32^ demonstrating that emotions are essential to motivate, guide, and facilitate rational action.^33^ Emotions are conceptualized as action tendencies;^34^ they instigate and regulate our actions (and inaction) in the social world and are thus involved in a much wider set of activities than the dramatic expressions with which they are typically associated. Trying to also account for subtle emotion linked to cognitive activities such as legal decision making, Barbalet distinguishes between ‘foreground’ and ‘background’ emotions.^35^ To be impartial and maintain an open mind, the judge needs to *feel* commitment, interest, and curiosity regarding her task, irritation over irrelevant information, pride in her skill in legal knowledge and encoding, and perhaps also aesthetic pleasure in the beauty of consistent categorization and the written judgment. A group of background emotions that is particularly relevant to our purposes here is that of epistemic emotions. These emotions are active in the acquisition and evaluation of knowledge and are thus entwined with cognitive activities.^36^ For example, a judge's certainty about her ruling is based both on her cognitive evaluation of the facts in the case and on her feeling of certainty linked to these evaluations.^37^ These emotions are not the foreground strong expressions that we commonly associate with irrational emotionality, but rather background calm emotions associated with subtle display. Foreground emotions disrupt purposive action; background emotions undergird it.^38^

Institutional interactions occurring in the courtroom also include strong foreground emotions, especially when stories are told during examinations of witnesses, victims, and defendants/litigants. People come to court when things go wrong, and their stories are often fraught with emotions such as anger, fear, and shame – emotions that legal professionals need to manage if they are to collect relevant evidence and move the procedure along.^39^ As lay narratives are emotionally laden, legal decision makers need to manage their own emotions to ensure an efficient process and to (re)produce trust in an objective and impartial outcome.^40^ Indeed, emotion management in court is an interactive process including both lay people and legal professionals.^41^

Both sets of emotional processes – the background emotions of legal reasoning and the foreground emotions of the legal process – are subjected to the ideal of judicial dispassion, influencing the way in which legal professionals work on their emotional responses to align to the norms of the legal setting and its feeling and display rules.^42^ Elsewhere, we have called the set of feeling and display rules that guide judicial conduct the ‘emotive‐cognitive judicial frame’,^43^ which includes the emotion management required to adjust feelings and concomitant bodily expressions to demonstrate objectivity and impartiality while acting in court. This judicial frame is a behavioural script that incorporates emotions within legal constraints, which require subtle emotional expressions and sensitivity to emotional cues so as to sustain the ideal of dispassion. To explore legal encoding in court, we need to examine the judicial frame and consider how its emotive‐cognitive norms guide the form that stories are allowed to take and how legal professionals listen to and evaluate them. In other words, we need to take into account the social landscape within which emotions arise in the legal realm.

An important process for understanding the emotional dynamics of guiding and evaluating stories in court is empathy.^44^ There are a wide range of definitions of empathy in the socio‐legal literature, but there seems to be agreement on three of the components involved: emotional attunement, perspective taking, and emotion management.^45^ Stories can promote emotional attunement and perspective taking in the form of the imagination of motives and intents of the people involved. Here, imagination serves to instigate a specific experiential perspective:^46^ that of the narrator who is telling the story, or that of a different character evoked in the narration. This implies that thinking and feeling become intertwined; emotions serve imagination by putting pressure on our representations so that we can imagine what is necessary for our epistemic needs and purposes.^47^ To give an example from the legal setting, a prerequisite for the crime of fraud is malicious intent. The judge needs to evaluate whether a defendant acted against a victim out of a desire for profit; in effect, she needs to imagine the defendant's perspective. To grasp the motivational state of the defendant, such as considering whether his actions constitute malice or care – competing hypotheses that are common in accusations of fraud perpetrated against older people – she needs to attune herself to both alternative stories.^48^ However, the judge's empathic attunement is guided by epistemic emotions such as scepticism, doubt, and curiosity – a topic that we develop below. When she imagines the environment and actions at stake, her assessment of the stories is shaped by what she finds intriguing or problematic, such as the fact that the accused falsely introduced himself as a relative of the victim. Details such as this can spur curiosity and orient perspective taking by focusing on certain streams of representation over others. The judge might imagine that the defendant's purpose in pretending to be a relative of the victim was to foster connections with people close to the elderly victim, his goal being to carry out his plan to take her money.

The capacity for empathic imagination is also linked to the structure of the narrative. Oatley links empathic perspective taking to the different components of a narrative: the ‘event structure’ (what happens in a story), the ‘discourse structure’ (instructions for interpreting the events), and the ‘suggestion structure’ (prompts for personal interpretations).^49^ The description of a crime in an indictment usually represents the event structure, which provides clues for interpretation of the actions at stake, but also directs attention to specific legal prerequisites. The event structure per se does not prompt empathic perspective taking. For that to occur, the story needs discourse and suggestion structures that can facilitate empathic imagination, such as during examinations in court.^50^

The third component of empathy, emotion management, is particularly relevant to legal decision making.^51^ Legal professionals need to manage their empathic responses when engaging in the process of narrative transformation, balancing attunement and distancing (proximation) to keep their focus on the relevant legal aspects. That is to say, perspective taking requires calibration while evaluating the probability of stories, the credibility of the statements, and the intent of the parties involved.^52^

To sum up, the process of narrative transformation and legal encoding takes place within an institutional‐emotional landscape in which legal professionals, oriented by an ideal of dispassion and objective justice, need to work on their emotions and empathic responses to translate accounts of events into legal categories.^53^ In the present article, we look at the transformation of narratives with an interactional eye, assuming that the way in which narratives are elicited and told influences how emotions come into play and are managed to enable legal encoding. Next, we combine these first two sections on narrative transformation, emotional processes, and emotion management ideals to explore how the telling and evaluation of stories in court are emotionally patterned.

## 4 LEGAL ENCODING AS EMOTION MANAGEMENT

In this section, we discuss the emotional dynamics of legal encoding linked to demarcation, fragmentation, and proximation. Our reasoning is backed up by emotion theories outlined in the previous section, but for the sake of clarity and practical application, we also use illustrations from Supreme Court rulings as well as observations and interview extracts from an ongoing international research project financed by the European Research Council (JUSTEMOTIONS 757625). One of the research questions within this larger project refers to the emotional dimensions of encoding subjective lay narratives in a legal case into objective legal categories. In the project, we investigate, using ethnographic methods, the emotive‐cognitive process of legal decision making in courts and prosecutors’ offices in two civil legal systems (Sweden and Italy) and two common legal systems (the US and Scotland). To obtain rich variation in emotional expectations and types of evidence, we compare legal professional decision making in cases of fraud, intimate partner violence, and homicide. We have shadowed prosecutors and judges while they prepare cases, observed them at hearings and deliberations, and interviewed them before and after hearings, gathering data on approximately 200 cases thus far. Shadowing entails following individuals during their workday, which gives opportunities to engage in continuous reflection on everyday practice.^54^ The fieldwork has included non‐participant observations of the professionals during trials and deliberations and participant observations during ‘in‐between’ situations (such as office work and breaks). In our non‐participant observations, we have focused on how stories are communicated and received by noting word choices, voice changes, body language, facial expressions, gestures, and glances. The continuous reflections made during shadowing have been supplemented with audio‐recorded interviews conducted using a semi‐structured interview guide. The combination of (participant and non‐participant) observations and interviews has created opportunities to reflect on the background emotional processes involved in narrative encoding. Linking questions to specific interactions and conversations close in time to their occurrence has encouraged recall of subtle emotional processes that likely would have been forgotten in a standalone interview. The analysis has focused on what parts of the narratives, at which stages in the process, were rendered legally (in)significant and on what seemed to be ‘triggers’ of engagement/disengagement. The analysis has followed an abductive process^55^ in that it has moved between the data and previous research, attending to how general legal principles of transforming narratives (in our terms, demarcation and fragmentation) are associated with emotional processes regulating emotional attuning (in our terms, proximation) and (dis)interest in stories. For the present article, our interest is in these ‘generic’ encoding processes, leaving a more fine‐grained analysis of potential cultural, systemic, and structural differences (such as gender, class, and ethnicity) to future publications. We use illustrations from Italy and Sweden, as in these civil legal systems professional judges (sometimes together with lay judges) make decisions on both guilt and sanction, thus allowing us to follow legal professionals’ active role in the transformation of stories into legal categories in the form of judgments. However, we argue that the techniques of demarcation, fragmentation, and proximation are relevant to both civil and common legal systems because they originate from the institutional legal procedures employed in most democratic societies. Furthermore, these techniques have universal emotional dynamics. For example, demarcation and fragmentation always delineate feelings of interest in stories, but the direction of that interest relates to the particular prerequisites and legal praxis within the legal system in which the story is being told. The empirical excerpts used here are followed by information on the type of case, type of data (interview/observation note), pseudonym, position, court, age of participant rounded to the nearest five years, and country.

### 4.1 Producing emotional distance from stories by demarcation and fragmentation

As Felstiner and colleagues argue, an important shift in the transformation of an everyday story into a law story is the changed emotional understanding of what happened, the blaming (‘He did not hurt me by mistake – he intended to hurt me’).^56^ While the transformation into disputes often heightens the emotions for the parties involved,^57^ it tends to subdue the emotions of legal professionals because encoding generally entails omitting the emotional content of an account.^58^ In this way, demarcation and fragmentation function, often implicitly, as emotion management strategies by distancing legal professionals from the emotional content and complexity of the stories that come before the court and moving them towards an interest in the parts of those stories that match with legal procedure and prerequisites. Emotional distance refers to not engaging with the coherent and potentially empathy‐inducing story at hand, but rather attuning oneself only to fragmented and demarcated parts of the stories that serve the purposes of categorization. Our intention here is therefore to link legal encoding to the emotional processes and emotion management strategies involved. As Tilly observes, ‘[c]odes emerge from the incremental efforts of organizations to impose order’,^59^ and as we argue, this order is emotionally patterned.

#### 4.1.1 Background emotions delineating (dis)interest in the case

Background emotions direct focus towards the task at hand and therefore become intimately entwined with cognition^60^ – in this case, the epistemic process of collecting and evaluating evidence for legal encoding. Demarcation and fragmentation are thus not only demands placed on the witnesses, victims, and defendants who tell their stories in court, but also constitute a professional demand to regulate focus and feelings of interest. This becomes evident by the lack of interest on the part of legal professionals when lay people giving their testimony do not follow the instructions to keep their focus on the trouble – that is, to keep the story within the required temporal boundaries. As described by a Swedish judge, ‘for the lay people involved, it is often the before, or even more often the after, [an event] that is relevant. But for us, that is totally, *totally* uninteresting!’ (interview, Asger, associate judge, district court, age 30, Sweden). Legal professionals should not only display interest in legal matters, but also demonstrate disinterest in the parts of stories that do not fall within their professional responsibility.^61^ This temporally narrow span of interest in a story thus preserves a focus on the details relevant to legal encoding, while keeping at bay a full‐blown emotional immersion in the stories told in court.

The emotional distance from stories becomes even more evident when we investigate not only temporal but also content demarcation and fragmentation of stories. Which parts of a story are considered interesting and which parts are considered irrelevant? On a methodological note, it is difficult to study non‐presence. When an event is described in a police report, many contextual and interactional details have already been omitted. Sometimes details emerge in trials unexpectedly, such as in one case in which a neighbour witness in an intimate partner violence hearing described, when she called the police, having heard the family dog bark along with the victim's cry (intimate partner violence, observation note, appeals court, Sweden). However, no one asked about the dog in the examinations. In law narratives, non‐participating characters are usually not regarded as important, whereas in everyday accounts their whereabouts would probably generate interest and curiosity, providing scope for a deeper inquiry. The demarcation and invisibility of these types of contextual and interactional features reduce complexity and details that can promote empathic perspective taking.^62^ Even though the trouble, such as a stabbing or a robbery, can evoke strong emotions in the participating actors, the lack of contextual and relational details leading up to and following the trouble limits the possibility for emotional attunement on the part of legal professionals. To use Oatley's distinctions,^63^ a focus on the rudimentary event structure of the context leading up to the trouble, together with a narrow interest span regarding the discourse structure for interpreting the events, limits the available prompts for personal interpretations (suggestion structure).

The span of interest concerning contextual and relational details is also linked to the particular prerequisites and legal praxis within the legal system in which the story is being told. After a deliberation in a homicide trial, Swedish appeals court Chief Judge Ruben criticizes the district court judgement's reference to a precedent from the supreme court. Swedish appeals courts can make new evaluations of both legal and factual elements depending on the motive of appeal, so the case can undergo a full scrutiny.^64^ The judgment states that, in some cases, the court can employ a method whereby it first evaluates the ‘combined weight of the evidence’ invoked by the prosecution, and

[i]f … the evidence is so strong that it is in itself sufficient for the evidentiary requirement to be met, the defendant's story and evidence supporting it must be examined. If what the accused is charged with is disproved, the prosecution must be dismissed.^65^

Judge Ruben argues that this method has been applied beyond its original intended scope and that the problem with first evaluating the combined evidence from one side is that it encourages a feeling of certainty too early in the deliberation (observation note, Ruben, chief judge, appeals court, age 60, Sweden). Instead, through the process of demarcation, judges should restrict their interest to fragmented pieces of evidence: ‘What is difficult about overall impressions is that it is not something we should have … An overall impression, you have to break it down and see: “What do the charges say?”’ (homicide, post‐hearing interview, Ruben, chief judge, appeals court, age 60, Sweden). Judge Ruben emphasizes the importance of demarcated interest in a case (‘you have to break it down and see’) so as not to risk feeling certain about a specific interpretation of events too early in the procedure. It is interesting to note that he openly reflects on how different methods of evaluating evidence influence the epistemic emotions at play. Embracing all of the evidentiary material in one take entails the risk of preventing curiosity about alternative interpretations. Ruben's open reflections on this emotional dynamic also indicate that the ideal of judicial dispassion is not necessarily threatened by background epistemic emotions.^66^

Demarcating content not only refers to limiting interest to the specific charges but also to how a story should be interpreted. Legal professionals need to demarcate objective data from non‐objective data, and this promotes emotional distance from the blurred, nuanced aspects of the story at hand. As we illustrate below, the importance of objective data or facts for law stories can reduce an evaluation of emotional experience, such as fear, to its behavioural display. The interview extract below refers to a case of stalking, in which the Italian appeals court Judge Roberto reflects on how he demarcates objective data from non‐objective data by describing how he made an objective evaluation of fear:^67^

You, *as a judge* [higher pitch], deduce things from a certain *declaration* [higher pitch]; for example, she tells you … ‘I was scared of him; I didn't want to see him’ because she … doesn't go where she used to; she has changed her phone number and tries to not contact him; when he calls, she says ‘You mustn't contact me’. She has closed her Facebook profile and ceased contact … So, you say ‘Her declaration is confirmed by this, this, this, and that …’ It's not that you deduce it by simply saying ‘I think she is telling the truth’. [Laughs] [*You need*] *the facts*!! [higher pitch] … How can you say someone makes you anxious when you write to them, asking them to come and see you and that you can't wait to see them for three days straight?! [He shouts, moves his shoulders forward, and gets closer to the interviewer] That is … [laughs] … it doesn't … stand up. (stalking, post‐hearing interview, Roberto, judge, appeals court, age 55, Italy)

A legal prerequisite for the crime of stalking in Italy is that the victim is afraid, so her emotion is of relevance to the legal encoding. In the quote, Judge Roberto speaks about the importance of evaluating fear only on objective facts. In his interpretation, certain behaviours indicate fear (changed phone number, no contact, closed Facebook profile), while others speak against fear and anxiety (writing to or wanting to see the accused). He argues that the victim's description of her emotion (‘I was scared of him; I didn't want to see him’) should not be considered, which implies that neither the victim's emotional display nor the judge's emotional experience should be used as clues in his evaluation.^68^ In Judge Roberto's view, the judge has no reason to attune himself emotionally to the victim's story; he should search for and tick off changes in behaviour.

#### 4.1.2 Irritation and frustration vs ease and relief

Another feature prominent in Judge Roberto's quote above is his passion for explaining what constitutes correct legal evaluation (indicated by his laughs, higher pitch, and shouting). The importance of demarcation and fragmentation to legal professionals can be seen in their irritation and frustration when too much information is introduced, or when they lose control over which aspects should be brought to the fore and which should be excluded from the law story. In written verdicts and procedural law, this is often depicted through reference to ‘unnecessary’, ‘irrelevant’, or ‘superfluous’ evidence.^69^ In the quote below, appeals court Judge Chiara describes her frustration with the defence lawyer's closing statement in a homicide case. His way of presenting the case as a full story, not just selecting the relevant parts, angers the judge because it distracts her attention from her task:

Unfortunately, *that lawyer* [higher pitch] … doesn't understand how things are. Because if you talk a lot, I'll listen to you, but after a while I'll get distracted, so you have to be able to highlight those five things *and emphasize those things* [higher pitch]! … [N]ot for example … whether the child was ill, and how high her temperature was – this doesn't interest us [angry tone]! (homicide, post‐hearing interview, Chiara, judge, appeals court, age 60, Italy)

Judge Chiara's interest and focus are restricted to ‘relevant’ issues, and when the defence lawyer embraces the whole story (‘the child was ill’), she gets ‘distracted’ and irritated (‘*emphasize those things*!’, ‘this doesn't interest us!’).^70^ This irritation directed at the defence lawyer fits with Abbott's argument about purification; the judge can tolerate that witnesses do not know the requirements for telling a law story, but legal professionals should be able to ‘purify issues for [her] consideration’.^71^ In Judge Chiara's view, the fuller picture becomes a distraction from her task of evaluating and encoding.

If irritation results from there being too much or ‘irrelevant’ detail in the law stories told in court, the ‘right amount’ of detail can instead give rise to contrasting feelings of relief or perhaps ease, as expressed by Swedish Judge Lydia:

[In this case] there were not so [sharp inhalations] many questions that were repeated, they focused on issues that were important. And it [exhales] gives a sense of relief in some way … And I don't think that it in any way disadvantaged the defendant [lowered voice] that too few questions were asked, not at all, but more just [laughter] we were not so run down [end laugh], it was easier to grasp the case, somehow. (homicide, post‐hearing interview, Lydia, judge, appeals court, age 45, Sweden)

As we can see from Judge Lydia's account, the demarcated story made her task easier (‘we were not so run down’), and she expressed relief at not having to sort through irrelevant details and a sense of ease or satisfaction (indicated by her exhale and laughter). As long ago as 1879, William James described ease as a rational feeling: ‘The transition from a state of puzzle and perplexity to rational comprehension is full of lively relief and pleasure.’^72^ In James’ (and Judge Lydia's) understanding, emotions of ease and relief do not threaten rational adjudication but rather promote it. Judge Lydia is careful to stress that her ease does not come at the expense of the defendant (articulating this potential worry with a lowered voice); on the contrary, she believes that the demarcated and fragmented story helped the court to make a decision (‘easier to grasp the case’).

In summary, demarcation and fragmentation are means of purifying stories for legal encoding, in effect distancing legal professionals from emotional attunement to the whole story at hand. The focus is on what happened (event structure) and to some extent the events’ meaning (discourse structure), whereas personal interpretations (suggestion structure) are often regarded as biasing or irrelevant, thus constraining empathic perspective taking. Maintaining emotional distance from the stories of a case does not mean that emotions are irrelevant to legal story building. As we have demonstrated, temporal deviations from the trouble and ‘too much’ context or relational detail lead to irritation, while a ‘proper’ amount of detail and a focus on behaviour to back up experience give rise to satisfaction or ease. That is to say, demarcation and fragmentation of law stories are sustained by the background emotions of irritation and ease/satisfaction. As is evident from their references to legal rules and prerequisites, legal professionals’ approach to stories, in contrast to the approach of jurors, is part of the professional skill that they developed in law school and have habituated with professional practice.^73^

### 4.2 Calibrating proximation

Legal decision making involves interpretation. When evaluating credibility, intent, and sanction, legal professionals must interpret people's experiences and therefore, we argue, they must calibrate proximation – that is, their level of closeness to the actual story and its emotional content. While our first two techniques, demarcation and fragmentation, function as indirect means to manage emotions when legal professionals regulate and steer how lay people's stories are told in court, our third dimension, proximation, refers to legal professionals’ deliberate techniques when listening to and evaluating stories – techniques used to maintain emotional distance from or proximity to the account at hand.

#### 4.2.1 To feel or not to feel

Legal professionals encounter stories of gruesome deeds, such as sexual abuse or homicide, and are required to listen to people who might have committed these crimes as well as those who have been the victims. This implies that the stories presented in court are often contradictory and competing; legal professionals need to take into account the fact that people lie or forget.^74^ To make a judgment, they must be critical when evaluating and determining which story, or which parts of a story, to believe. In theory, there are two opposite ways of managing this tension: trying to be equally interested in and empathically attuned to all stories that they hear,^75^ or being equally sceptical and doubtful of all stories, such as in the following example, in which Swedish Judge Leo employs empathic distance:

To feel or not to feel? It's as if I register what this person tells me, and then when I've heard all the other witnesses, too, I can feel ‘how this thing really is’. I mean, I kind of presuppose that before I've heard everyone, I can't know … if it actually happened this or that way. I think I allow myself to feel some doubt until it's clear to me that everyone has told the exact same story, so there is no doubt about it anymore – it did happen the way he said it did. (interview, Leo, associate judge, district court, age 35, Sweden)

Judge Leo uses doubt as a filter through which he listens to the stories. With doubt as his point of departure, the recounted stories are received and evaluated from a distance, representing possible – not necessarily real – scenarios. Doubt becomes a method for avoiding preconceptions and thus an emotion management strategy to remain impartial during the presentation of evidence in the trial. If proximation were a scale ranging from full empathic immersion to full distance, Leo would represent the latter. However, in real life, emotional response to a story is not always so neatly apportioned.

In cases of intimate partner violence, Italian jurisprudence allows for a conviction based only on the victim's statements, if they meet specific requirements:

The declarations of the injured person can be legitimately placed on their own as a basis for the assertion of criminal responsibility of the accused, after verification accompanied by suitable motivation, of the subjective credibility of the declarant and the intrinsic reliability of his story, which in this case must be more penetrating and rigorous than other witness declarations.^76^

In practical terms, the judge must evaluate whether the victim's story is coherent, detailed, clear, and without contradictions.^77^ In the following example, Italian Judge Aurora reflects on her way of approaching a story of intimate partner violence. She does not position herself on the scale of full immersion versus full distance, but instead focuses on her own immediate emotive‐cognitive response to a story:

I always go there with an empty mind … I say ‘Let's see now what they tell us’ [laughs]. Of course, when I see the person who made a report … *I listen to them* [higher pitch] … [I]n this case … I therefore realize a little … eh … that the story is standing! It is likely, coherent … she [the victim] told it in a way that convinced me … with pain … I, right now, *don't* [higher pitch] have any great doubts … that things went as the woman told us. (intimate partner violence, post‐hearing interview, Aurora, judge, tribunal,^78^ age 55, Italy)

Judge Aurora does not stress either doubt about or immersion in the story, but instead reflects on her own response in an open‐ended way, articulating both factual and emotional clues (the story is ‘likely’, ‘coherent’, and told ‘with pain’). In contrast to Judge Roberto, whom we encountered in a previous section when he defended a strict focus on behaviour, Judge Aurora also takes the victim's emotional display (told ‘with pain’) into account in her interpretation. She does not find her own siding with one party problematic. To understand this, we need to introduce temporality, as mentioned by both Judge Leo and Judge Aurora. They link their emotional response to the phase of the trial (Judge Leo: ‘before I've heard everyone, I can't know’; Judge Aurora: ‘I, right now, *don't* have any great doubts’). To ensure that they evaluate all evidence or versions of stories with equal interest or doubt, judges are required to not make up their mind until all evidence has been presented and all stories have been heard.^79^ It is therefore imperative for legal decision makers to regulate their feelings of certainty.^80^ As we have seen, when we add temporality as a criterion for evaluating law stories, one‐sided empathic immersion is not necessarily problematic.

#### 4.2.2 Critically evaluating certainty

In another intimate partner violence case described below, Italian Judge Rossella indeed fully immerses herself in the victim's story, and her original stance is that the defendant is guilty. However, this initial conviction is just a starting point for further inquiry: ‘I am convinced that the defendant has done these things, but I need to look for an objective validation from other parties, since I cannot rely on the victim’ (intimate partner violence, pre‐hearing interview, Rossella, judge, tribunal, age 60, Italy). In this case, the victim has a psychiatric diagnosis that could cast some doubt on the credibility of her statements. After complementary examinations of the victim's physician, it becomes clear that the judge cannot use the victim's accusations as grounds for a guilty verdict. Judge Rossella's initial trust and emotional investment in the victim's story come to the fore in her description of this development as a failure: ‘[with an agitated voice] We have the objective data as she states them, but we cannot give an evaluation of credibility. So, everything *collapsed* on me! [higher pitch] Apart from the things that are surely objective’ (intimate partner violence, post‐hearing interview, Rossella, judge, tribunal, age 60, Italy). She has invested emotionally in this story, and finding out that she cannot use it as evidence evokes strong emotions (‘everything collapsed on me!’).

One could assume that such one‐sided empathic immersion would prove contradictory to an impartial evaluation (compare Judge Ruben's earlier statement about the dangers of certainty). However, this depends on how she manages her feeling of certainty. Certainty is an emotion associated with the cessation of interest in further inquiry.^81^ Once one has reached certainty, one does not want to go out into uncertainty again. For judges, however, the statutory requirement to keep an open mind might make them more willing to challenge or critically evaluate their own certainty. Judge Rossella's belief in or certainty about the victim's story did not necessarily evaporate, but she did not use her feelings as grounds for her judgment. They rather provided an entry point for critical scrutiny:

I … [hesitates] … at the very end, I think something has really happened, because he [the defendant] is surely an aggressive guy. We can see that from the assault he committed against the doctor … from the fact that he damaged the doors of the hospital, where she [the victim] was hospitalized … from the assaults against the administrators, and from all his numerous criminal records. Yes, the sensation is that something has happened … but we have not been able to prove it … I feel at ease, because, anyway, it is always better, between the two, to acquit … wrongly … rather than to convict … wrongly … here we are talking about years in prison [laughs] … so, I didn't really feel like [convicting the defendant]! (intimate partner violence, post‐hearing interview, Rossella, judge, tribunal, age 60, Italy)

Judge Rossella acquitted the defendant and felt good about it, even though she was still certain of his guilt. Her emotional investment – taking pride in making an impartial and objective decision (‘we have not been able to prove it’) – took precedence over her empathic attunement and belief in the victim's story and, in the end, proved emotionally rewarding (‘I feel at ease’). Rather than remaining uncertain until all of the evidence was presented, Judge Rossella felt certain early on in the process, but was prepared to challenge and dispute her feelings of certainty, to scrutinize them from the perspective of statutes and procedural rules.

To sum up, for legal professionals proximation involves an array of strategies to manage and put emotions to work when listening to and evaluating stories in court. They can employ one emotional strategy throughout by approaching stories with complete empathic immersion (proximity) or doubt (distance) to remain uncertain until all stories have been told and all evidence has been presented. Alternatively, they can calibrate their proximation throughout the process by articulating and reflecting on their emotional responses (like Judge Rossella), continuously being prepared to challenge their certainty or judge despite their feelings of certainty (like Judge Aurora), when what they think happened differs from what can be proved.

## 5 CONCLUSION

For the legal decision maker, breaking up coherent narratives is seen as central to procedurally correct decision making. Demarcation places narratives within narrow and strictly defined boundaries, while fragmentation separates legally relevant from irrelevant aspects with respect to both how and when the stories are told. The goal, of course, is correct categorization. As we have argued, this encoding process promotes emotional distance from stories by channelling feelings of interest and curiosity towards the demarcated and fragmented details of the stories, while blocking the temporal, content, and relational information necessary for empathic perspective taking and emotional attunement. The professional focus on categorization is further backed up by feelings of ease, relief, and satisfaction when stories are presented according to legal requirements, versus disinterest and irritation when ‘too much’ detail is introduced.

However, legal evaluation and decision making also require attuning oneself to the stories told in court, and we have introduced the concept of proximation to highlight legal professionals’ deliberate regulation of empathic perspective taking and emotional attunement to law stories. We argue that this attunement is regulated through the epistemic emotions of doubt and certainty.^82^ To evaluate contradictory stories critically, legal professionals need to strategically regulate their feelings of doubt, challenge or go against their feelings of certainty, and reflect on their emotional attunement to the stories.^83^

Maintaining emotional distance from stories in court is in accordance with the ideal of judicial dispassion,^84^ but, as we have seen, this ideal does not paint the full picture. First, the effort to maintain emotional distance from the often strong foreground emotions of law stories is backed up by more subtle background emotions linked to encoding.^85^ Second, legal interpretation of and deliberation on stories explicitly demand emotions of scepticism, doubt, and certainty. As indicated by the judges’ articulation of these emotions in our empirical illustrations, they might not be included, at least to the same extent, in the dispassionate ideal. In contrast to the often foreground emotions of fear or anger that flourish in law stories, these epistemic emotions are linked to cognitive processes of rational deliberation and thus do not constitute a threat to the rational ideal. Though the illustrations used in the present article refer to judges, these techniques also apply to prosecutorial decision makers, as they are essential in forming the law stories presented in court and they make pre‐trial and plea decisions (depending on the legal system) based on their encoding evaluations.

Encoding represents the legal system's ‘incremental efforts to impose order’,^86^ and the patterning of this order is therefore pivotal to understanding the social structure of legal evaluation and decision making. In applied terms, the process of encoding highlights the constraints put on the witnesses, victims, or defendants/litigants telling their stories in court.^87^ More directly, the legal transformation of everyday stories into law stories pinpoints how gaps can emerge between a common‐sense understanding and a legal understanding of a case. Our intention has not been to conduct a coherent empirical analysis of all relevant emotional processes involved in the legal transformation of stories into legal categories, but to develop conceptual tools for further empirical research. The three transformative techniques presented here require further empirical validation to explore how they as well as other techniques vary across countries, courts, or types of cases in terms of how narratives are approached and employed in the decision‐making process. Such a venture would provide further theoretical insights into epistemic emotional processes and, in the longer run, validate the implementation of more stringent and deliberate tools for legal professionals’ management of emotions in narrative transformation and legal encoding.

## Footnotes

1 D. Haraway, ‘Situated Knowledges: The Science Question in Feminism and the Privilege of Partial Perspective’ (1988) 14 *Feminist Studies* 575.

2 J. J. A. Shaw, *Law and the Passions: Why Emotion Matters for Justice* (2020).

3 J. Bruner, *Making Stories: Law, Literature, Life* (2002).

4 S. L. Winter, ‘The Cognitive Dimension of the Agon between Legal Power and Narrative Meaning’ (1989) 87 *Michigan Law Rev*. 2225, at 2228.

5 M. Turner, *The Literary Mind: The Origins of Thought and Language* (1998) 4.

6 N. Pennington and R. Hastie, ‘A Cognitive Theory of Juror Decision Making: The Story Model’ (1991) 13 *Cardozo Law Rev*. 519; R. H. Conley and J. M. Conley, ‘Stories from the Jury Room: How Jurors Use Narrative to Process Evidence’ in *Studies in Law, Politics and Society*, ed. A. Sarat (2009) 25.

7 R. Lempert, ‘Telling Tales in Court: Trial Procedure and the Story Model’ (1991) 13 *Cardozo Law Rev*. 559; S. Cammiss, ‘“He Goes Off and I Think He Took the Child”: Narrative (Re)Production in the Courtroom’ (2006) 17 *King's Law J*. 71; F. Di Donato, *The Analysis of Legal Cases: A Narrative Approach* (2020).

8 In a bench trial, the judge decides alone because the defendant has waived his right to a jury. It is used in the United States (US) in 10–15 per cent of criminal cases and 30–35 per cent of civil cases, but equivalent forms where judges rule alone or in panels exist in several common legal systems. T. A. Mauet, ‘Bench Trials’ (2002) 28 *Litigation* 13.

9 Preliminary studies have demonstrated that professional judges approach the stories told in court in different ways than lay arbiters do, particularly in relation to emotional dimensions. E Wessel et al., ‘Credibility of the Emotional Witness: A Study of Ratings by Court Judges’ (2006) 30 *Law and Human Behavior* 221.

10 T. A. Maroney, ‘The Persistent Cultural Script of Judicial Dispassion’ (2011) 99 *California Law Rev*. 629.

11 See for example A. J. Wistrich et al., ‘Heart Versus Head: Do Judges Follow the Law or Follow Their Feelings?’ (2014) 93 *Texas Law Rev*. 855.

12 Bruner, op. cit., n. 3.

13 M. Djikic et al., ‘Reading Other Minds: Effects of Literature on Empathy’ (2013) 3 *Scientific Study of Literature* 28.

14 W. L. F. Felstiner et al., ‘The Emergence and Transformation of Disputes: Naming, Blaming, Claiming …’ (1980) 15 *Law and Society Rev*. 631. For example, in examinations legal professionals ask questions to identify the prerequisites pertinent to a particular type of crime or other legal category.

15 A. Abbott, ‘Status and Status Strain in the Professions’ (1981) 86 *Am. J. of Sociology* 819, at 824.

16 C. Tilly, *Why?* (2008).

17 Bruner, op. cit., n. 3, p. 48.

18 M. Bladini, *I Objektivitetens Sken: En Kritisk Granskning av Objektivitetsideal, Objektivitetsanspråk och Legitimeringsstrategier i Diskurser om Dömande i Brottmål* (*In the Semblance of Objectivity: A Critical Review of Objectivity Claims and Legitimation Strategies in Criminal Trial Discourses*) (2013); Bruner, op. cit., n. 3; K. L. Scheppele, ‘Foreword: Telling Stories’ (1989) 87 *Michigan Law Rev*. 2073; Di Donato, op. cit., n. 7.

19 Bruner, id.

20 W. A. Beach, ‘Temporal Density in Courtroom Interaction: Constraints on the Recovery of Past Events in Legal Discourse’ (1985) 52 *Communications Monographs* 1, at 8.

21 S. Bogira, *Courtroom 302: A Year behind the Scenes in an American Criminal Courthouse* (2005).

22 Scheppele, op. cit., n. 18, p. 2094.

23 Bladini, op. cit., n. 18; A Kjus, *Stories at Trial* (2010); M. Kelman, ‘Interpretive Construction in the Substantive Criminal Law’ (1981) 33 *Stanford Law Rev*. 591.

24 Felstiner et al., op. cit., n. 14.

25 Kelman, op. cit., n. 23.

26 N. G. Fielding, ‘Lay People in Court: The Experience of Defendants, Eyewitnesses and Victims’ (2013) 64 *Brit. J. of Sociology* 287, at 300.

27 For example, the principles of immediacy and orality refer to the need to ‘produce’ the evidence before the court – the judge should have direct and oral contact with witnesses instead of deciding on papers; hearsay rules prohibit testimony quoting persons who are not in court (so‐called first‐hand witness); witnesses are not allowed to give statements of opinion – they should only tell what they have seen or heard, without adding personal judgments about it.

28 T. Booth, ‘“Cooling Out” Victims of Crime: Managing Victim Participation in the Sentencing Process in a Superior Sentencing Court’ (2012) 45 *Aus. & New Zealand J. of Criminology* 214.

29 T. A. Maroney, ‘Emotional Regulation and Judicial Behavior’ (2011) 99 *California Law Rev*. 1481; Wistrich et al., op. cit., n. 11.

30 L. Laudan, *Truth, Error, and Criminal Law: An Essay in Legal Epistemology* (2006).

31 B. Lange, ‘The Emotional Dimension in Legal Regulation’ (2002) 29 *J. of Law and Society* 197, at 215.

32 T. A. Maroney and J. J. Gross, ‘The Ideal of the Dispassionate Judge: An Emotion Regulation Perspective’ (2014) 6 *Emotion Rev*. 142; S. Roach Anleu and K. Mack, *Judging and Emotion: A Socio‐Legal Analysis* (2021); S. A. Bandes (ed.), *The Passions of Law* (1999); S. Bergman Blix and Å. Wettergren, *Professional Emotions in Court: A Sociological Perspective* (2018).

33 J. Barbalet, *Emotion, Social Theory, and Social Structure: A Macrosociological Approach* (2001); S. Bergman Blix, ‘Making Independent Decisions Together: Rational Emotions in Legal Adjudication’ (2022) 45 *Symbolic Interaction* 50.

34 N. H. Frijda, *The Emotions* (1986).

35 Barbalet, op. cit., n. 33, ch. 2.

36 R. de Sousa, ‘Epistemic Feelings’ (2009) 7 *Mind and Matter* 139; A. Morton, ‘Epistemic Emotions’ in *The Oxford Handbook of Philosophy of Emotion*, ed. P. Goldie (2010) 385.

37 Bergman Blix, op. cit., n. 33.

38 J. Barbalet, ‘Emotions beyond Regulation: Backgrounded Emotions in Science and Trust’ (2011) 3 *Emotion Rev*. 36; J. Barbalet, ‘Science and Emotions’ (2002) 50 *The Sociological Rev*. 132.

39 Bergman Blix and Wettergren, op. cit., n. 32; Roach Anleu and Mack, op. cit., n. 32; J. A. Scarduzio, ‘Maintaining Order through Deviance? The Emotional Deviance, Power, and Professional Work of Municipal Court Judges’ (2011) 25 *Management Communication Q*. 283.

40 Roach Anleu and Mack, id.; Bergman Blix and Wettergren, id.

41 Booth, op. cit., n. 28; Fielding, op. cit., n. 26.

42 A. R. Hochschild, *The Managed Heart: Commercialization of Human Feeling* (1983).

43 Bergman Blix and Wettergren, op. cit., n. 32, p. 22.

44 S. A. Bandes, ‘Empathetic Judging and the Rule of Law’ (2009) *Cardozo Law Rev. De Novo* 133; M. Del Mar, ‘Judging Virtuously: Developing an Empathic Capacity for Perceptual Sensitivity’ (2014) 5 *Jurisprudence* 196; M. C. Nussbaum, ‘Emotion in the Language of Judging’ (1996) 70 *St. John's Law Rev*. 23.

45 S. Bergman Blix, ‘Different Roads to Empathy: Stage Actors and Judges as Polar Cases’ (2019) 1 *Emotions & Society* 163.

46 M. Balcerak Jackson, ‘On the Epistemic Value of Imagining, Supposing, and Conceiving’ in *Knowledge through Imagination*, eds A. Kind and P. Kung (2016) 41.

47 A. Morton, *Emotion and Imagination* (2013).

48 M. Del Mar, ‘Imagining by Feeling: A Case for Compassion in Legal Reasoning’ (2017) 13 *International J. of Law in Context* 143; Morton, id.; de Sousa, op. cit., n. 36.

49 K. Oatley, *Such Stuff as Dreams: The Psychology of Fiction* (2011).

50 Bergman Blix, op. cit., n. 45.

51 Id.

52 Å. Wettergren and S. Bergman Blix, ‘Empathy and Objectivity in the Legal Process: The Case of Swedish Prosecutors’ (2016) 17 *J. of Scandinavian Studies in Criminology and Crime Prevention* 19.

53 E. Goffman, *Frame Analysis: An Essay on the Organization of Experience* (1974); Bergman Blix and Wettergren, op. cit., n. 32.

54 B. Czarniawska, *Shadowing and Other Techniques for Doing Fieldwork in Modern Societies* (2007).

55 S. Timmermans and I. Tavory, ‘Theory Construction in Qualitative Research: From Grounded Theory to Abductive Analysis’ (2012) 30 *Sociological Theory* 167.

56 Felstiner et al., op. cit., n. 14.

57 Id.

58 Bergman Blix and Wettergren, op. cit., n. 32.

59 Tilly, op. cit., n. 16, p. 125.

60 Barbalet, op. cit. (2002), n. 38; Barbalet, op. cit. (2011), n. 38.

61 T. Parsons, ‘The Professions and Social Structure’ (1939) 17 *Social Forces* 457.

62 J. Zaki, ‘Empathy: A Motivated Account’ (2014) 140 *Psychological Bull*. 1608.

63 Oatley, op. cit., n. 49.

64 Since 2008, Sweden has adopted ‘a modern trial’ model whereby the appeals courts, instead of redoing the whole trial, watch video recordings from the examinations made in the district court.

65 National Judicial Archive (NJA), 2015, s. 702, at <https://lagen.nu/dom/nja/2015s702> (author's translation from Swedish). The judgment referred to a case of attempted homicide. Based on a piece‐by‐piece evaluation of the evidence, the appeals court had acquitted the defendant. When the supreme court decided to instead evaluate the combined weight of the evidence, the defendant was found guilty.

66 It is also interesting to note that, in contrast to previous theorizing on jury story modelling, professional legal decision makers integrate legal rules and prerequisites in their first encounter with stories, not as a second step, as the ‘story model’ implies. According to the story model, jurors make decisions in three steps: first, they evaluate evidence through story construction; second, they study the decision alternatives (verdict categories) by learning their attributes or elements; third, they place the story into the best‐fitting category. Pennington and Hastie, op. cit., n. 6.

67 Just as in Sweden, Italian appeals courts can decide on both factual and legal elements of a case depending on the reasons for appeal. In contrast to Sweden, however, the appeals trials review most of the cases only on paper; they do not make new oral examinations of witnesses unless it is considered ‘absolutely necessary’. Code of Criminal Procedure (CPP) (1998), Art. 603, at <https://www.altalex.com/documents/news/2014/09/03/appello>.

68 One problem with this demarcation strategy is that emotional experience and behavioural display do not necessarily correspond exactly in the way in which Judge Roberto assumes that they do. No emotional experience of any kind by necessity leads to a specific expression or behaviour. Emotions are conceptualized as action *tendencies*; they make us ready to act, but do not dictate our actions. Frijda, op. cit., n. 34.

69 See for example CPP, op. cit., n. 67, Art. 190.

70 From the defence lawyer's perspective, giving a fuller account can have the purpose of showing the client that you take the case seriously and will bring forward all potentially relevant aspects in the defence.

71 Abbott, op. cit., n. 15, p. 824.

72 W. James, ‘The Sentiment of Rationality’ (1879) 4 *Mind* 317, at 317.

73 L. Flower, ‘The (Un)Emotional Law Student’ (2014) 6 *International J. of Work Organisation and Emotion* 295; Å. Wettergren and S. Bergman Blix, ‘Prosecutors’ Habituation of Emotion Management in Swedish Courts’ (2021) *Law & Social Inquiry* 1; S. Goodrum and M. C. Stafford, ‘The Management of Emotions in the Criminal Justice System’ (2003) 36 *Sociological Focus* 179.

74 P. Brooks, ‘Narrative in and of the Law’ in *A Companion to Narrative Theory*, eds J. Phelan and P. J. Rabinowitz (2005) 415.

75 Del Mar, op. cit., n. 44; Nussbaum, op. cit., n. 44.

76 Cassazione Penale, 21/09/2018, No. 52999, at <https://canestrinilex.com/risorse/censure‐specifiche‐e‐obbligo‐di‐motivazione‐in‐appello‐cass‐5299918/>. See also the equivalent in the Swedish precedent: ‘An entirely credible statement from the plaintiffs can, in conjunction with what has otherwise emerged in the case – e.g. on the plaintiff's conduct after the event – be sufficient for a conviction.’ NJA, 2009, s. 447, at <https://lagen.nu/dom/nja/2009s447>.

77 Cassazione Penale, 20/03/2019, No. 12250, at <https://www.doppiadifesa.it/wp‐content/uploads/2019/04/Bacio‐sul‐collo‐e‐violenza‐sessuale_Cassazione‐penale‐sez.‐III‐sentenza‐20.03.2019‐n.‐12250.pdf>.

78 Tribunals (in other words, trial courts) are the courts of first instance in Italy. At the tribunals, cases are decided by a single judge or a panel of three judges, depending on the crime.

79 Roach Anleu and Mack, op. cit., n. 32, p. 65. Keeping an open mind is also required earlier on in the processing of a case through the legal system. The police and the prosecutors, too, should not make up their mind before all relevant evidence has been collected and evaluated.

80 Bergman Blix, op. cit., n. 33.

81 de Sousa, op. cit., n. 36.

82 Id.

83 M. Holmes, ‘The Emotionalization of Reflexivity’ (2010) 44 *Sociology* 139.

84 Maroney, op. cit., n. 10.

85 See also Bergman Blix and Wettergren, op. cit., n. 32.

86 Tilly, op. cit., n. 16, p. 125.

87 For a similar argument related to an earlier stage of the process, see Felstiner et al., op. cit., n. 14.

